# Mass Detection in Viscous Fluid Utilizing Vibrating Micro- and Nanomechanical Mass Sensors under Applied Axial Tensile Force

**DOI:** 10.3390/s150819351

**Published:** 2015-08-06

**Authors:** Ivo Stachiv, Te-Hua Fang, Yeau-Ren Jeng

**Affiliations:** 1 Department of Mechanical Engineering, National Kaohsiung University of Applied Sciences, Kaohsiung 80778, Taiwan; E-Mail: fang@kuas.edu.tw; 2 Institute of Physics, Czech Academy of Sciences, Prague 18221, Czech Republic; 3 Advanced Institute of Manufacturing with High-tech Innovations (AIM-HI), National Chung Cheng University, Chiayi County 62102, Taiwan; 4 Department of Mechanical Engineering, National Chung Cheng University, Chiayi County 62102, Taiwan

**Keywords:** mass resonator sensors, cantilever mass sensors, resonant frequency, carbon nanotube, viscous fluid, beam under tension, mass detection in fluid

## Abstract

Vibrating micro- and nanomechanical mass sensors are capable of quantitatively determining attached mass from only the first three (two) measured cantilever (suspended) resonant frequencies. However, in aqueous solutions that are relevant to most biological systems, the mass determination is challenging because the quality factor (*Q*-factor) due to fluid damping decreases and, as a result, usually just the fundamental resonant frequencies can be correctly identified. Moreover, for higher modes the resonance coupling, noise, and internal damping have been proven to strongly affect the measured resonances and, correspondingly, the accuracy of estimated masses. In this work, a technique capable of determining the mass for the cantilever and also the position of nanobeads attached on the vibrating micro-/nanomechanical beam under intentionally applied axial tensile force from the measured fundamental flexural resonant frequencies is proposed. The axial force can be created and controlled through an external electrostatic or magnetostatic field. Practicality of the proposed technique is confirmed on the suspended multi-walled carbon nanotube and the rectangular silicon cantilever-based mass sensors. We show that typically achievable force resolution has a negligibly small impact on the accuracy of mass measurement.

## 1. Introduction

Micro-/nanosized beams are the fundamental component used in nanotechnology for detection of various physical quantities including pressure, force [1], quantum state [2], spin [3], thin film mechanical properties [4], and molecule masses [5]. Particularly, the vibrating micro-/nanomechanical mass sensors possess the ultrahigh sensitivity, excellent selectivity, and operating frequencies up to several gigahertzes with the extraordinary controllability via optomechanical or electromechanical coupling, and, finally, they enable real-time mass measurement with the capability of reaching the ultimate limits of mass detection [6,7]. These devices usually measure shift of the flexural resonant frequencies caused by the attached molecule. Resonant frequencies decrease when the molecules or nanoparticles are attached on the resonator surface and the value of the frequency shift depends on the attached masses and the positions of attachment [8,9]. It has been shown that by measuring multiple vibrational modes (two for suspended and three for cantilever), single particle mass can be unambiguously determined [10]. Recently, this approach was successfully implemented in a single-protein real-time mass detection in a vacuum [7].

In general, flexural vibration of micro-/nanosized beams including those used as mass sensors can be described by a well-known Euler-Bernoulli beam equation. This equation, however, predicts accurately just the fundamental resonant frequencies, while for higher modes, the accuracy of predicted resonances reduces due to either the internal friction losses [11] or the existence of coupling between in- and out-plane flexural, torsional, and longitudinal motions [12]. Furthermore, in aqueous solutions that are relevant to most biological systems, dissipation due to surrounding media dominates, causing a large decrease of the quality factor (*Q*-factor) and, consequently, usually just the fundamental resonant frequencies can be correctly identified [13]. Exceptionally, for very specific beam configurations, the first two flexural modes can be detected [14]. As a result, in gases and liquids including the physiological solutions, the attached molecules are evaluated from the measured shift of either the flexural fundamental mode by considering the uniform distribution of added masses over the entire resonator length [15] or the flexural and longitudinal/torsional fundamental modes [16]. The former method does not account for the exact particle or molecule attachment position(s), therefore, the quantitative mass determination of even a single molecule by one is not possible. The latter method is capable of a single mass detection in fluid but, nevertheless, it is challenging to simultaneously determine fundamental flexural and longitudinal/torsional vibrational modes.

In this paper, we extend our previous works [10,16,17] to report the modified technique capable of mass determination in viscous fluid without the necessity of knowing the surrounding fluid density and viscosity. This technique utilizes the measurement of the fundamental resonant frequencies of the micro-/nanomechanical mass sensor under different values of the intentionally applied axial tensile force, which is created and controlled either via an external electrical/electromagnetic field [5,17] or mechanically [18]. In addition, due to the importance in real application use, we show that the commonly achievable force measurement errors, e.g., inaccuracy in the estimation of the electrical force, have a negligibly small impact on the extracted mass values.

## 2. Theory of Mass Determination in Fluid by the Axially Loaded Micro-/Nanomechanical-Based Mass Sensors

### 2.1. Statement of the Problem and the Basic Theory of the Mass Sensor

**Figure 1:**
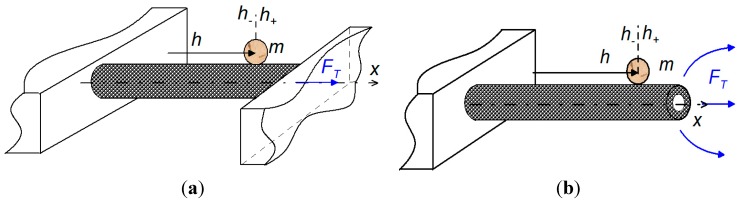
A schematic representation of the nanomechanical mass sensor under axial tensile force with an attached particle in (**a**) suspended and (**b**) cantilever configurations.

In this section, we provide theoretical ground and application limits of the present technique of mass extraction from the measured fundamental resonant frequencies. It is worth noting that the present method of mass extraction is primarily applicable to the mass sensors operating in vacuum, gases, and low viscosity fluids including aqueous solutions such as water-glycerol solutions, and for cases where the mass of attached nanoparticles, nanobeads, or molecules dominates the adsorption. It means that the flexural rigidity change caused by the attached molecules has a negligibly small impact on the sensor frequency response. For example, the usual biomolecules have the diameter of ~10 nm and the elastic modulus of *~*1 GPa, resulting in the flexural rigidity change of order *O* (10^−12^) Nm^2^ [19]. Since flexural rigidity of the commonly used microsized mass sensors is usually *O* (10^−7^) Nm^2^ [20], therefore, in this case, the adsorption is indeed dominated by the molecule mass. However, when the sensor thickness or diameter has a comparable dimension as the binding biomolecule, nanoparticle, or nanobead, then the frequency response caused by the molecule adsorption is dominated by molecule mass and its stiffness. Qualitative measurement of the attached masses in nanosized mass sensors operating in air with account for the adsorption effects can be found in works of Wasisto *et al*. [21,22]. Furthermore, suspended and cantilever micro-/nanomechanical mass sensors under applied axial tensile force can be modeled as an elastic beam under applied constant tensile force (see Figure 1). The beam and the surrounding fluid must also satisfy the following criterion:

(a) The beam length, *L*, exceeds its dominant cross-section scale *W*_D_, e.g., for a rectangular (circular) case *L* >> *W* (*D*_out_), where *W* (*D*_out_) is the resonator width (outer diameter);
(b) The beam is made from isotropic linearly elastic solid material(s), and the shear deformations, rotary inertia, and internal friction effects are negligible;
(c) The cross-section of the beam is uniform over its the entire length;
(d) The beam vibrational amplitude is essentially smaller than any of its length scale;
(e) Dissipative effects due to internal friction losses are negligibly small compared to those caused by the surrounding fluid (this assumption holds mainly just for the lower vibrational modes [11]);
(f) Flexural resonant frequencies are distinct (it is always satisfied for the fundamental mode, whereas for the higher modes the coupling between flexural and torsional/longitudinal modes can be realized [12]);
(g) Fluid surrounding beam is incompressible in nature;
(h) The mass and size of the attached nanobead is essentially smaller than the mass of the beam itself, *i.e.*, the attached mass does not change the beam mode shape and has negligibly small influence on the fluid-structure interaction.

We must also emphasize here that for beams with low *Q*-factors (*Q* ≤ 1), the vibrational amplitude would fall in the low signal-to-noise ratio and, correspondingly, the resonant frequencies might not be correctly identified. Fortunately, many experimental studies revealed that a large number of the commonly used micro-/nanomechanical resonators operating in gaseous solutions have *Q* >> 1 and in aqueous solutions *Q* > 1 [13,14,15,23], hence enabling the correct identification of the fundamental resonance frequencies, e.g., even for nanowire- and carbon nanotube-based resonators, the quality factor in deionized water (DI water) can be still higher than five [13,14]. Additionally, for many nano-/micromechanical beams operating in air or aqueous solutions, the impact of the dissipative part of the hydrodynamic force on the resonator motion in close vicinity to the resonance peak is negligibly small [23,24,25,26,27]. Then, in the area of the resonance peaks, the modes of the beam can be considered to be uncoupled and, at the resonance frequency, the hydrodynamic function (hydrodynamic load) Γ(ω) can be evaluated in absence of the dissipative effects, *i.e.*, the real part of Γ(ω) dominates the motion. We note here that the fundamental theoretical grounds needed to predict the flexural motion of the beam submerged in viscous fluid are given in Appendix A, whereas for a detailed derivation and analysis of the hydrodynamic function, the reader is further referred to [24,25,26,27,28,29]. In this case, the simple resonant frequencies in viscous fluid are

(1) $$f_{\text{R}}=\frac{f_{V}}{\sqrt{1+\frac{\pi \rho_{l}W_{\text{D}}^{2}}{4\rho A}\Gamma_{r}(\omega_{\text{R}})}}$$

where ρ*A* is the linear density of the beam, ρ*_l_* is the surrounding fluid density, Γ*_r_* is the real component of the hydrodynamic function [24], ω_R_ = 2π*f*_R_, and *f_V_*_,*n*_ are just the simple resonant frequencies of the beam in a vacuum given by

(2) $$f_{V}=\frac{{\gamma_{V}}^{2}}{2\pi }\sqrt{\frac{EI}{\rho A}}$$

where *EI* is the beam flexural rigidity and γ*_V_*^2^ is spectrum of the dimensionless resonant frequencies obtained as a positive root of the appropriate transcendental equation, which, for suspended configuration of the beam under tension (Figure 1a), reads

cosh*q*_1_ cos*q*_2_ − 1 − *b*^2^/(2*q*_1_*q*_2_) sinh*q*_1_ sin*q*_2_ = 0 (3a)

and, similarly, for the cantilever beam (Figure 1b) transcendental equation is given by

(3b) $$(1+\frac{b^{4}}{2{\left(q_{1}q_{2}\right)}^{2}})\cosh q_{1}\;\cos q_{2}+1+\frac{b^{2}}{2q_{1}q_{2}}\;\sinh q_{1}\;\sin q_{2}=0$$

where *q*_1,2_ = [±*b*^2^/2 + (*b*^4^/4 + γ*_V_*^4^)^1/2^]^1/2^, *b* = (*F_T_L*^2^/*EI*)^1/2^ is the dimensionless tension parameter [30], and *F_T_* is an applied axial tensile force.

**Figure 2:**
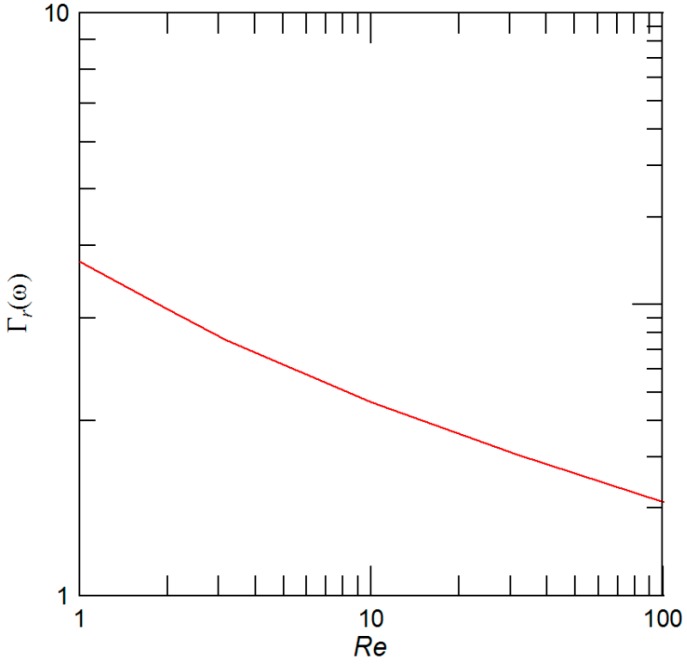
Dependency of the real part of hydrodynamic function, Γ*_r_*, on the Reynolds number, *Re*.

Resonant frequencies in fluid predicted by Equation (1) generally depend on the beam frequency in vacuum, sensor dimension, fluid density, and viscosity through Γ*_r_* [24,27]. In addition, the validity of Equation (1) has been confirmed by several experimental [13,14,23] and numerical [25,26] studies. Moreover, it has been shown in [24] and is also illustrated in Figure 2 that the real part of the hydrodynamic function depends weakly on frequency through the Reynolds number, *Re* = ρ*_l_*ω*W_D_*^2^/(4μ*_l_*), where μ*_l_* is the fluid viscosity and ω is the angular frequency. Then, within the usual frequency shifts of the mass resonator sensors (~*O* (10^2^–10^4^) Hz), the Γ*_r_* can be regarded as constant without affecting the accuracy of mass extraction from data as is going to be illustrated later in Section 3 on the cantilever-based mass sensors made of silicon, which are operating in DI water.

The mass of the attached nanobeads, particles, or molecules, *m*_∑_, where *m*_∑_ = $\sum_{i=1}^{N}m_{\text{i}}$ and *N* stands for the number of attached masses, is essentially smaller than the mass, *M*, of the sensor (beam) itself, *i.e.*, *m*_∑_ << *M* [10,16]. Thus, each of the attached nanobeads enters into the problem equation, *i.e.*, Equation (A1) given in Appendix A, only through the following so-called “matching conditions”

(4) $$u(h_{i–},\;t)=u(h_{i+},\;t),\;\frac{\partial u(h_{i-},\;t)}{\partial x}=\frac{\partial u(h_{i+},\;t)}{\partial x},\frac{\partial^{2}u(h_{i-},\;t)}{\partial x^{2}}=\frac{\partial^{2}u(h_{i+},\;t)}{\partial x^{2}}\;EI\;\left[\frac{\partial^{3}u(h_{i-},\;t)}{\partial x^{3}}\;-\;\frac{\partial^{3}u(h_{i+},\;t)}{\partial x^{3}}\right]-F_{T}\;\left[\frac{\partial u(h_{i-},\;t)}{\partial x}\;-\;\frac{\partial u(h_{i+},\;t)}{\partial x}\right]\;=\;m_{i}\frac{\partial^{2}u(h,\;t)}{\partial t^{2}}$$

where subscript *i* stands for *i*-th attached mass, and *h*_+_ and *h*_–_ stand for the location to the right and to the left of the attached mass as shown for in Figure 1. To find the explicit form of equation describing the dimensionless resonant frequencies of the beam under an arbitrary value of applied axial tensile force that accounts for *N* masses, we employ the procedure primarily developed to solve the natural frequencies of longitudinally oscillating beams with *N*-piecewise constant mechanical properties [31]. Firstly, we separate the governing beam equation, *i.e.*, Equation (A1) in Appendix A with accounting for *F*_dr_ = *F*_hd_ = 0, into *N* + 1 sections with respect to the attached particle masses. Secondly, at the location of each particle the particular matching conditions given by Equation (4) are applied, and then the problem is closed by the appropriate boundary conditions, *i.e.*, the clamped-free end for the cantilever configuration and the clamped-clamped end for the suspended one. And finally, solving the *N* + 1 algebraic equations yields the required transcendental equation, which, for a suspended configuration, takes the following form

(5a) $$\cosh q_{1}\;\cos q_{2}–1–b^{2}/(2q_{1}q_{2})\;\sinh q_{1}\;\sin q_{2}–\sum_{i=1}^{N}\varepsilon_{i}\sum_{j=1}^{m\Sigma }\frac{q_{1}^{3}H_{\text{B}}(q,h_{j})}{2(q_{1}^{2}+q_{2}^{2})}$$

and for a cantilever beam, the equation reads

(5b) $$(1+\frac{b^{4}}{2{\left(q_{1}q_{2}\right)}^{2}})\;\cosh q_{1}\;\cos q_{2}+1+\frac{b^{2}}{2q_{1}q_{2}}\;\sinh q_{1}\;\sin q_{2}–\sum_{i=1}^{N}\varepsilon_{i}\sum_{j=1}^{m\Sigma }\frac{q_{1}^{5}A_{0}H_{\text{C}}(q,h_{j})}{2q_{2}^{2}(q_{1}^{2}+q_{2}^{2})}=0$$

where ε = *m*/*M* is the mass ratio and coefficients *A*_0_, *H_B_*(*q*, *h*), and *H*_C_(*q*, *h*) are given in Appendix B. Equation (5) describes the resonant frequency spectrum of the mass sensor under an arbitrary value of applied axial tensile force that accounts for the attached mass

### 2.2. Procedure of the Single (Multiple) Mass Determination by Means of the Vibrating Suspended- and Cantilever-Based Mass Sensors under Intentionally Applied Axial Tensile Force

Here, based on the analytical results, we propose a technique of single and multiple mass determination utilizing the frequency shift measurement of only the fundamental flexural resonant frequencies of micro-/nanosized mass sensors under different intentionally applied axial tensile force.

Following the approach given in work of Stachiv *et al.* [10] and accounting for Equations (3) and (5), the frequency shift of the mass sensor under *F_T_* caused by the attached masses can be obtained as

∆*f*/*f*_0_ = 2ε_∑_α_∑_(h_j_^*^, γ_0_) (6)

where ∆*f* = *f*_0_ – *f*, *f*_0_ and *f* are unloaded and loaded by *N* nanobeads resonant frequencies, ε_∑_ = *m*_∑_/*M*, α_∑_(h_j_^*^, γ_0_) is the position function given in Appendix B, h_j_^*^ = *h_j_*/*L* are the dimensionless attachment positions, *i.e.*, *j* ∈ {1, …, *m_N_*}, *L* is the sensor length, and γ_0_^2^ are the dimensionless resonant frequencies of the unloaded sensor obtained by solving Equation (3). Importantly, Equation (6) allows us to separate the effect of mass and position on the resonant frequency shift. Furthermore, for a given vibrational mode, the mass and its position of attachment, the frequency shift, and the position function α(h_j_^*^, γ_0_) depend only on the applied axial tensile force, *i.e.,* γ_0_ varies with *F_T_*. Therefore, the frequency shift of the beam without tension can be directly obtained from Equation (6) by letting *F_T_* = 0, and it is given by

∆*f*_B_/*f*_0B_ = 2ε_∑_α_∑_(h_j_^*^, γ_0B_) (7)

where α(h_j_^*^, γ_0__B_) are given in Appendix B and γ_0B_ = 4.73 and 1.875 for suspended (Figure 1a) and cantilever (Figure 1b) configurations, respectively.

**Figure 3:**
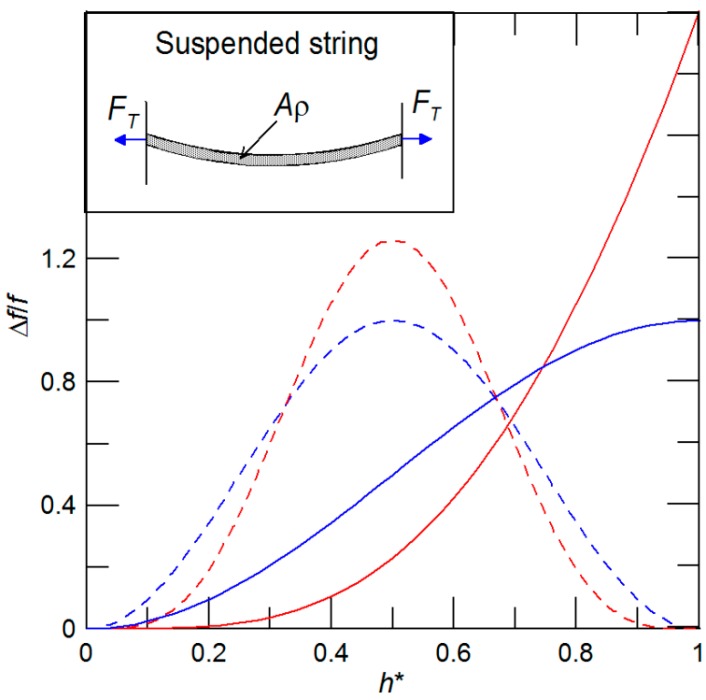
Dependency of the frequency shift of the fundamental mode on the dimensionless position h^*^ for a given mass ratio and two limiting cases: beam (red line) and string (blue line) in suspended (dash line) and cantilever (solid line) configurations, respectively. Figure inset shows the sketch of pure string in a suspended configuration.

Similarly, in the limit of large axial forces, the flexural rigidity *EI* can be neglected and, consequently, the mass sensor vibrates as a “pure” string. For an illustration we provide a sketch of the suspended string in the inset of Figure 3. The matching conditions of the string are obtained from Equation (4) for *EI* = 0 are as follows

(8) $$u(h_{i–},\;t)=u(h_{i+},\;t),\;F_{T}\;\left[\frac{\partial u(h_{i+},\;t)}{\partial x}-\frac{\partial u(h_{i-},\;t)}{\partial x}\right]=m_{i}\frac{\partial^{2}u(h,\;t)}{\partial t^{2}}$$

and the corresponding frequency shift of string that accounts for the attached mass reads

∆*f*_s_/*f*_s0_ = ε_∑_α_∑_(h_i_^*^, λ_0_) (9)

where for the suspended configuration α(h^*^, λ_0_) = (–1) sin(λ_0_h^*^) sin[λ_0_ (1 – h^*^)]/cosλ_0_ and λ_0_ = π, and for the cantilever configuration α(h^*^, λ_0_) = sin(λ_0_h^*^) cos[λ_0_(1 – h^*^)]/sinλ_0_ and λ_0_ = π/2.

For a given mass ratio ε_∑_, the frequency shift of the beam without tension by Equation (7) and string by Equation (9) differ from each other only through the position function of 2α (see Figure 3). Then we can easily conclude that the frequency shift of the beam under tension can be realized only between the beam and string cases, where the exact shift values depend on the magnitude of applied axial tensile force. In general, an increase of *F_T_* shifts ∆*f*/*f*_0_ from the beam without tension towards a pure string. Additionally, in our previous work, we have shown that for a fundamental mode of the suspended mass sensor, the position function starts to significantly deviate from α(h^*^, γ_0B_) at values of *b* > 5 [17]. Similarly, by performing computations over a large number of *F_T_*_,_ we found that for the cantilever configuration, the position function starts to notably deviate from α(h^*^, γ_0B_) at *b* > 3. Therefore, by detecting the frequency shifts of the mass sensor under different *F_T_*, the attached mass can be unambiguously determined. It is worth noting that there can only be one resonant frequency shift measurement in the case of the cantilever (suspended) mass sensor performed for *b* ≤ 3 Equation (5), while others must be carried out for *b* > 3 Equation (5).

Interestingly, the alternative and widely used technique of mass extraction utilizes the approximate solution obtained by the Raleigh-Ritz method [6,7,8,9]. For an approximate solution, the frequency shift of the beam under applied axial tensile force can be expressed as

*f*/*f*_0_ = (1 + ε_∑_Y_∑_^2^(*x*))^−1/2^ (10)

where the mode shape of the cantilever mass sensor under *F_T_* is given by

Y(*x*) = sinh*q*_1_*x* − *G*_C_ cosh*q*_1_*x* − *q*_1_/*q*_2_ sin*q*_2_*x* + *G*_C_ cos*q*_2_*x* (11a)

and for the suspended mass sensor, the corresponding mode shape reads

Y(*x*) = sinh*q*_1_*x* − *G*_B_ cosh*q*_1_*x* − *q*_1_/*q*_2_ sin*q*_2_*x* + *G*_B_ cos*q*_2_*x* (11b)

where *G*_C_ = (*q*_1_^2^sinh*q*_1_ + γ_0_^2^ sin*q*_2_)/(*q*_1_^2^cosh*q*_1_ + *q*_2_^2^cos*q*_2_) and *G*_B_ = (sinh*q*_1_ − *q*_1_/*q*_2_ sin*q*_2_)/(cosh*q*_1_ − cos*q*_2_). We must emphasize here that in Equation (10), just the normalized mode shapes are used, *i.e.*, $\int_{0}^{1}{\text{Y}}^{\text{2}}(x)dx$ = 1. Importantly, the frequency shift given by Equation (6) can be rewritten in the following way

*f*/*f*_0_ = 1–2ε_∑_α_∑_(h_j_^*^, γ_0_) (12)

**Figure 4:**
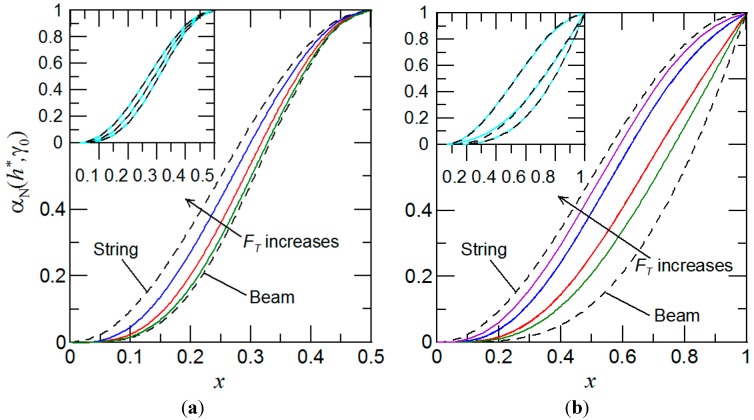
Variation of α_N_(h^*^, γ_0_) for different values of *F_T_*, where α_N_(h^*^, γ_0_) = α(h^*^, γ_0_)/α_max_(h^*^, γ_0_) and α_max_(h^*^, γ_0_) is the maximum value of α for (**a**) suspended and (**b**) cantilever configurations, respectively. The green color represents α_N_(h^*^, γ_0_) at *b* = 5 (for other colors the *b* values differ for suspended and cantilever cases). Insets present the comparison between mode shapes (dashed line), *Y*_N_(*x*) = [Y(*x*)/Y_max_(*x*)]^2^, and position functions (solid line), α_N_(h^*^, γ_0_) = α(h^*^, γ_0_)/α_max_(h^*^, γ_0_), for different values of *F_T_*. Y_max_(*x*) stands for the maximum value of the mode shape.

Then, comparing Equations (10) and (12), and neglecting the higher order terms, *i.e.*, *O*(ε^2^), it is possible to express the position function α(h_j_^*^, γ_0_) through the mode shape Y(*x*), also illustrated in the inset of Figure 4, as follows

α(h_j_^*^, γ_0_) = (1/4) Y^2^(*x*) (13)

It is important to note that cantilever mass sensor expressions for α(h_j_^*^, γ_0_) are cumbersome (see Equations (B1) and (B3) in the Appendix B), hence, in this case, the mode shape could be preferably used to extract the mass values.

Furthermore, it can be concluded from Figure 3 and Figure 4 that estimating the mass of each of the attached nanobeads requires the measurement of at least two different resonant frequency shifts. Now, let us suppose that there is a population of *N* masses attached on the micro-/nanomechanical mass sensor. Then, to determine the attached masses, *P* shifts of the fundamental resonant frequencies under different axial prestress forces are detected, where *P* ≥ 2*N*. Mathematically, Equations (6) and (10) take the following matrix forms

(14) $$U\rho \bar{d}=\bar{R_{\text{ω}}}$$

where **ρ** $\bar{d}$ = [ε1ε2⋮εN], $\bar{d}$ is the *N*-elements unitary vector, and for the perturbation technique represented by Equation (6) **U** = [ $\bar{\alpha_{1}},\bar{\alpha_{2}},...,\bar{\alpha_{N}}$], $\bar{\alpha_{N}}$ = $\left[\begin{array}{c} \sum_{j=1}^{m\sum }\alpha (h_{j}^{*},\gamma_{01})\\ \sum_{j=1}^{m\sum }\alpha (h_{j}^{*},\gamma_{02})\\ \vdots \\ \sum_{j=1}^{m\sum }\alpha (h_{j}^{*},\gamma_{0P}) \end{array}\right]$, $\bar{R_{\text{ω}}}$ = $\left[\begin{array}{c} \text{Δ}f_{1}/(2f_{01})\\ \text{Δ}f_{2}/(2f_{02})\\ \vdots \\ \text{Δ}f_{P}/(2f_{0P}) \end{array}\right]$, whereas for the energy approach given by Equation (10) **U** = [$\bar{{\text{y}}_{1}},\bar{{\text{y}}_{2}},...,\bar{{\text{y}}_{N}}$], $\bar{{\text{y}}_{N}}$ = $\left[\begin{array}{c} \sum_{j=1}^{m\sum }{\text{Y}}_{\text{1}}^{\text{2}}(x_{h_{j}})\\ \sum_{j=1}^{m\sum }{\text{Y}}_{\text{2}}^{\text{2}}(x_{h_{j}})\\ \vdots \\ \sum_{j=1}^{m\sum }{\text{Y}}_{P}^{\text{2}}(x_{h_{j}}) \end{array}\right]$, $\bar{R_{\text{ω}}}$ = $\left[\begin{array}{c} f_{1}/f_{01}\\ f_{2}/f_{02}\\ \vdots \\ f_{P}/f_{0P} \end{array}\right]$.

Then, we seek positions h_j_^*^and the relative mass changes ε*_i_* that satisfy Equation (14). Briefly, the expected attachment positions are obtained numerically by the method of least squares and afterwards, for obtained h_j_^*^, the desired mass ratios ε*_i_* can be found. Thus, finally, for the known mass of the nano-/micromechanical mass sensor, the desired individual masses of *N* attached nanoparticles, nanobeads, and/or molecules can be unambiguously estimated. Furthermore, for a cantilever configuration, this technique allows one to simultaneously determine the mass and position of the attached nanoparticles. It is due to the fact that for the cantilever, the position function and the mode shape have unique values for each h_j_^*^ (see Figure 3 and Figure 4b). For the suspended beam, the position function and mode shape are symmetric at the middle part of beam, *i.e.*, h^*^ = 0.5, as evident from Figure 3 and Figure 4a. Thus, as a result, the same values of position function and mode shape exist for two different attachment positions, except for the particle attachment at h^*^ = 0.5. Correspondingly, in case of a single particle mass measurement, two shifts of the cantilever fundamental resonant frequencies under different *F_T_* are needed to evaluate the particle mass and its position. We must emphasis here that for cantilever mass sensors that are not modulated by *F_T_* resonant frequencies, *i.e.*, the mass sensor vibrates as beam only, three consecutive resonant frequencies are needed to determine the mass and its position [10].

Importantly, our proposed technique of mass measurement from the fundamental mode of an axially loaded mass sensor differs from the previously reported technique of multiple particle mass determination from the several consecutive resonant frequencies of the cantilever beam [9]. The numerical solution of Equation (14) is, however, identical with the one given in work of Dohn *et al.* [9]; thus, the reader is referred to their work for a detailed discussion on the numerical solution of the present problem.

It is worth noting that the accuracy of mass evaluation by means of the axially loaded mass sensors depends on the uncertainties in force measurement. In this work, we use the perturbation technique to derive the relative error in mass determination *d*ε caused by the small uncertainties in force measurement represented through *d*α, *df*, and *df*_0_, and, as a result, the equation yields

(15) $$d\varepsilon /\varepsilon \;(=dm/m)\approx \frac{f}{f_{0}}\left(\frac{df_{0}}{f_{0}-f}\right)\;-\;\frac{df}{f_{0}-f}\;-\;\frac{d\alpha }{\alpha }$$

Resonant frequencies *f* and *f*_0_ are generally proportional to *b* as *K*_1_*b*^2^ + *K*_2_*b*, where *K*_1,2_ are the coefficients depending on the resonator configuration and the applied axial force, e.g., for string regime *K*_1_ = 0 [17]. For the cantilever (suspended) configuration of the mass sensor and *b* ≤ 3 Equation (5), the variation of Δα/α is negligible and the accuracy of mass measurement depends only on Δ*f* and Δ*f*_0_. Typical force resolution of the micro-sized resonators is of sub-piconewton (*O* (0.1 pN)) [32] (in vacuum and for a low temperature, the force accuracy of attonewtons can be reached [33]), and for nano-sized resonators, the detectible forces are of sub-femtonewton [34].

## 3. Results and Discussion

To begin, we recall the known fact that flexural oscillations of the majority of micro-/ nanosized beams, including those used as mass sensors, are realized and controlled by an external electrical or electromagnetic field, which creates a constant axial force [5,17,35,36,37,38]. In order to verify the practicality of the proposed technique, the suspended multi-walled carbon nanotube-based mass sensor (MWCNT) of density 2.1 g/cm^3^, elastic moduli 1.15 TPa, and of length 11.4 μm with outer and innermost diameters of 15 nm and 3 nm, respectively, which is loaded by a mass of *m* = 122 ag, *i.e.*, ε ≈ 0.03, with an attachment position at *h* = 5.6 μm, is considered. The uncertainty error in predetermined forces is 0.1 pN, three orders of magnitude higher than the common force resolution of the nanomechanical resonators [32]. The fundamental resonant frequencies of MWCNT under *F_T_* = 3.65 (*b* = 12.89) and 16.7 nN (*b* = 27.58) and are 5.403 MHz ± 0.06 kHz (γ_0_ ≈ 7.021) and 10.305 MHz ± 0.03 kHz (γ_0_ ≈ 9.697), respectively. The attached molecule mass causes the frequency shift (Δ*f*) of 178.3 ± 0.1 and 320.3 ± 0.15 kHz, respectively. It results in the frequency shift ratio [Δ*f*_1_/*f*_01_]/[Δ*f*_2_/*f*_02_] of 1.062 with an error of *O* (10^–4^), where subscripts 1 and 2 stand for *F_T_* = 3.65 and 16.7 nN. Now, accounting for the dependence of α(h^*^, 7.021)/α(h^*^, 9.697) (it is obtained by solving Equation (B4) given in Appendix B), the value of 1.062 agrees with the possible attachment positions at h^*^ = 0.49 or 0.51. For α (0.49/0.51, 7.021) = 0.57, the corresponding ratio of ε ≈ 0.03 is found yielding, for a known mass of MWCNT, the desired molecule mass of *m* ≈ 122 ag. As can be seen from this example, the commonly achievable uncertainties in force measurement [32,33,34] do not affect the accuracy of determined mass.

Now, we turn our attention to the mass sensor operating in a specific environment, e.g., air, gaseous, and aqueous solutions. Particularly, we consider a silicon cantilever (ρ = 2.33 g/cm^3^, *E* = 169 GPa) of dimension 200 μm (*L*), 20 μm (*W*), and 1 μm (*T*-thickness) loaded by a mass of *m* = 187 pg (ε = 0.01) at position *h* = 195 μm under *F_T_* = 1 (*b* = 0.38) and 100 μN (*b* = 3.77) operating in DI water. Again, as in the previous example, the corresponding resonant frequencies are 4.59 and 10.38 kHz, and the frequency shifts caused by attached mass are 85 and 175 Hz, yielding the frequency ratio of 1.098. Then, accounting for the dependency of α(h^*^, γ_0_) obtained by solving Equation (B3), the possible attachment position h^*^ = 0.97 is found, and the mass ratio of ε ≈ 0.01 can be retrieved.

It is important to note that for mass sensors operating in a viscous fluid the frequency shift caused by the attached mass significantly decreases, making its measurement highly challenging, e.g., the above-considered silicon cantilever Δ*f* in a vacuum is of one order higher than the one in DI water. In general, fluid damping decreases the mass sensitivity, whereas the axial tension causes its increase. Due to the practical importance in mass measurement, we now analyze an improvement of the mass sensitivity of the resonant-based mass sensor submerged in fluid caused by the applied axial tensile force. Accounting for Equation (1), the dimensionless mass sensitivity is obtained in the following way

(16) $$S\approx ({\gamma_{0}}^{2}–\gamma^{2})/\varepsilon \left[1-P\left(\frac{\rho_{l}}{\rho }\right)\;\Gamma_{r}(f_{\text{R},\;n})\right]$$

where *P* = (π/8)(*W*/*T*) or 1/2 for rectangular or circular resonator cross-sections. Table 1 and Table 2 present the achievable mass sensitivity of the silicon cantilever mass sensors loaded by mass of ε = 0.01 at its free end, *i.e.*, h^*^ = 1. From Equation (16) and Table 1 and Table 2, the following important conclusions can be drawn: (i) for a given ε and the sensor dimension, the mass sensitivity in a specific environment decreases; and (ii) the mass sensitivity can be significantly enhanced either by the tensile axial force represented through the tension parameter *b* or by increasing sensor thickness. Thus, in fluid, the required mass sensitivity, *i.e.*, sensitivity needed to detect the target molecule, can be achieved by optimizing the sensor dimension and by applying the axial tensile force, whose exact value can be estimated from Equations (3), (5) and (16).

**Table 1:** The achievable mass sensitivities of the cantilever mass sensors made of silicon with *L* = 200 μm, *W* = 30 μm, and thicknesses *T* = 1, 2, and 4 μm in a vacuum and air-loaded by a mass of ε = 0.01 at h^*^ = 1 as function of an applied axial tensile force.

	Vacuum	Air			
	(all cases)	*T* = 0.5 μm	*T* = 1 μm	*T* = 2 μm	*T* = 4 μm
*b* = 0	6.84	6.68	6.76	6.80	6.82
*b* = 2	10.54	10.30	10.42	10.48	10.51
*b* = 5	15.96	15.61	15.78	15.87	15.91
*b* = 8	20.01	19.59	19.80	19.91	19.96

**Table 2:** The achievable mass sensitivities of the cantilever mass sensors made of silicon with *L* = 200 μm, *W* = 30 μm, and thicknesses *T* = 1, 2, and 4 μm in DI water and 24% glycerol-water solution (GWS) of ρ*_l_* = 1.053 g/cm^3^ and μ*_l_* = 1.984 Pa·s loaded by a mass of ε = 0.01 at h^*^ = 1 as function of an applied axial tensile force.

	DI Water/24% GWS			
	*T* = 0.5 μm	*T* = 1 μm	*T* = 2 μm	*T* = 4 μm
*b* = 0	0.33/0.31	0.63/0.60	1.16/1.11	1.98/1.90
*b* = 2	0.52/0.49	0.98/0.94	1.80/1.72	3.08/2.96
*b* = 5	0.80/0.76	1.53/1.46	2.79/2.67	4.75/4.58
*b* = 8	1.03/0.98	1.96/1.87	3.58/3.42	6.07/5.85

Present conclusions are also valid for sensors of other cross-sectional areas even it is not shown here. Additionally, for sensors with non-uniform cross-sectional areas, the methodology of mass extraction from the fundamental mode is identical with the one derived for sensors with uniform cross-sectional areas. Nevertheless, for measurements in aqueous solutions, the frequency shift given by Equation (1) must be recalculated for the exact sensor dimension. Thus, in the case of three-dimensional structures, the numerical methods must be employed in order to determine required resonant frequency shifts in fluid with and without accounting for the attached masses.

## 4. Conclusions

We proposed a technique capable of quantitative mass determination from only measured fundamental resonant frequencies of the micro-/nanomechanical mass sensors. This technique benefits from the mode shape and frequency shift changes caused by the variation of the intentionally applied axial tensile force. In addition, since it utilizes measurements of the sensor’s fundamental resonant frequencies with and without attached mass and the computation of the frequency ratio Δ*f*/*f*, the knowledge of fluid density and viscosity is not required, and the impact of noise and damping on accuracy of the results is also minimized. The commonly achievable uncertainties in force measurement have been proven to have a negligibly small impact on the extracted mass values.

Our findings can find an application in real-time single and multiple mass detection in gaseous and aqueous solutions, where usually just the fundamental resonant frequency can be correctly identified. In a vacuum, measurement of the several consecutive resonant frequencies under intentionally applied axial tensile force helps to significantly increase the number of measured attached masses and, as a result, enables real-time mass spectrometry in a vacuum.

## Appendix A

Since oscillations of beams submerged in fluid have been extensively studied and, consequently, theoretical models that are capable of accurately predicting resonances in fluid have been already developed [23,24,25,26,27,28,29,39], in this Appendix, we provide only a fundamental theoretical ground of beams submerged in viscous fluids that is needed to derive Equation (1). To begin, we recall the known fact that the flexural motion of the mass sensor under applied axial tensile force submerged in viscous fluid can be described by the following differential equation

(A1) $$\rho A\frac{\partial^{2}u(x,\;t)}{\partial t^{2}}+EI\;\frac{\partial^{4}u(x,\;t)}{\partial x^{4}}-F_{T}\frac{\partial^{2}u(x,t)}{\partial x^{2}}=F_{\mathrm{dr}}(x,\;t)+F_{\mathrm{bd}}(x,\;t)$$

where *F*_dr_(*x*, *t*) and *F*_hd_(*x*, *t*) are driving and hydrodynamic forces per unit length, respectively. Then, scaling the spatial variable *x* with respect to *L* (for readers’ convenience hereafter the spatial variable *x* refers to the scaled quantity) and taking the Fourier transform of Equation (A1) yields

(A2) $$\alpha^{2}\frac{\partial^{4}U(x,\;\omega )}{\partial x^{4}}-\beta^{2}\frac{\partial^{2}U(x,\;\omega )}{\partial x^{2}}-\rho A\omega^{2}U(x,\;\omega )=F_{\mathrm{dr}}(x,\omega )+F_{\mathrm{bd}}(x,\;\omega )$$

where *U* = $\int_{-\infty }^{\infty }u(t)e^{-i\omega t}dt$, ω is the frequency variable, *i* is a complex number, α = (*EI*/*L*^4^)^1/2^ is the flexural rigidity coefficient, β = (*F*_T_/*L*^2^)^1/2^ is the tension coefficient [40], *F*_dr_(*x*, ω) is the force responsible for beam actuation and *F*_h__d_(*x*, ω) reads

*F*_h__d_(*x*, ω) = (π/4)ρ*_l_W*_D_^2^ω^2^Γ(ω)*U*(*x*, ω) (A3)

Plugging Equation (A3) into Equation (A2) yields

(A4) $$\frac{\partial^{4}U(x,\;\omega )}{\partial x^{4}}-b^{2}\frac{\partial^{2}U(x,\;\omega )}{\partial x^{2}}-{\left[G(\omega )\right]}^{4}U(x,\omega )=\alpha^{2}F_{\mathrm{dr}}(x,\omega )$$

where

(A5) $$G(\omega )={\gamma_{\text{V}}}^{2}\sqrt{\frac{\omega }{\omega_{\text{V}}}}{\left(1+\frac{\pi \rho_{l}W_{\text{D}}^{2}}{4A\rho }\Gamma (\omega )\right)}^{1/4}$$

The flexural oscillations of an arbitrary beam operating in viscous fluid can be predicted by solving Equation (A4). The general solution of Equation (A4) for an arbitrary driving force can be obtained by, for instance, the eigenfunction expansion technique, and this equation reads

(A6) $$U(x,\;\omega )=\sum_{n=1}^{\infty }f_{n}(\omega )Y_{n}(x)$$

where *Y_n_*(*x*) are the normalized mode shapes given by Equation (11) that are known to form an orthonormal basic set [40], *i.e.*, $\int_{0}^{1}Y_{m}(x)Y_{n}(x)dx=0$ for *m* ≠ *n*, and *f_n_*(ω) are the functions of frequency that are determined by substituting Equation (A6) into Equation (A4) and applying the orthonormal properties of *Y_n_*(*x*), and they read

(A7) $$f_{n}(\omega )=\frac{\alpha^{2}\int_{0}^{1}F_{\text{dr}}(x^{\prime },\omega )Y_{n}(x^{\prime })dx^{\prime }}{{\gamma_{V}}^{4}-G{\left(\omega \right)}^{4}}$$

For many nano-/micromechanical beams submerged in fluid, the dissipative effect of fluid is much smaller than the added mass due to surrounding fluid (see for example [6,13,23]). Then, the changes in *G*(ω) near resonance peaks are governed by *O*(ω^2^), *i.e.*, the variation of Γ(ω) depends weakly on *O*(ω^−1/2^) [31], and, correspondingly, in the area of the resonance peak, the hydrodynamic function can be considered a constant and can be evaluated at the resonance frequency in the absence of dissipative effects. In the area of the resonance peak *G*(ω) = γ*_V_* $\sqrt{\frac{\omega }{\omega_{V}}}$ ${\left(1+\frac{\pi \rho_{l}W_{\text{D}}^{2}}{4A\rho }\left[\Gamma_{r}(\omega )+i\Gamma_{i}(\omega )\right]\right)}^{1/4}$ and for Γ*_r_* >> Γ*_i_*, the resonant frequencies given by Equation (1) can be obtained.

## Appendix B

Here, the coefficients *H_C_*(*q*, *h*) and *H*_B_(*q*, *h*) used in Equation (5), and the position functions α(*h_j_*^*^, γ_0_) and α(*h_j_*^*^, γ_0__B_) required to extract the attached masses from frequency shifts by Equations (6) and (7) are provided. The explicit form of coefficient *H_C_*(*q*, *h*) is

*H*_C_(*q*, *h*) = *A*_1_^4^*F*(*qh*) + *A*_1_^3^[cosh*q*_1_*h* sin(*q*_2_(1 − *h*)) cos*q*_2_ + sinh*q*_1_*h* sinh(*q*_1_(1 − *h*)) sin*q*_2_] + *A*_1_^2^[*F*(*q*) − *F*(*q*(1 − *h*)) − cosh*q*_1_*h* cos(*q*_2_(1 − *h*)) sin*q*_2_] + *A*_1_*G*(*q*(1 − *h*)) + *A*_1_^5^*A*_2_ sin*q*_2_[cos*q*_2_ + sin*q*_2_*h* sin(*q*_2_(1 − *h*))] + *A*_1_^4^*A*_2_[*F*(*q*(1 − *h*)) − (sinh*q*_1_ + sin*q*_2_) cos(*q*_2_(1 − *h*)) cos*q*_2_*h*] + *A*_1_^3^*A*_2_[*G*(*q*) + cosh(*q*_1_(1−*h*)) sin*q*_2_*h* cos*q*_2_] − *A*_1_^2^*A*_2_ [*F*(*qh*) + sinh*q*_1_ sin*q*_2_*h* sin(*q*_2_(1 − *h*)) + cosh(*q*_1_(1 − *h*)) cos*q*_2_*h* sin*q*_2_] + *A*_1_*A*_2_*G*(*qh*) − cosh*q*_1_*h* cosh(*q*_1_(1 − *h*)) sin*q*_2_ + (*b*/*q*_2_)^2^ {*A*_1_^2^[*F*(*qh*) − sinh(*q*_1_*h*) cosh(*q*_1_(1 − *h*)) cos*q*_2_] + *A*_1_[*G*(*q*) + *G*(*qh*) − *G*(*q*(1 − *h*))] + *A*_1_^3^*A*_2_[*G*(*q*(1 − *h*)) − cosh*q*_1_ cos(*q*_2_*h*) sin(*q*_2_(1 − *h*))] + *A*_1_^2^*A*_2_[*G*(*q*(1−*h*)) + *G*(*qh*) − *G*(*q*)] + *A*_1_*A*_2_[cosh*q*_1_ sin(*q*_2_*h*) cos(*q*_2_(1 − *h*)) − *G*(*qh*)] + cosh(*q*_1_*h*) sinh(*q*_1_(1 − *h*)) cos*q*_2_ − *F*(*q*(1 − *h*))}
(B1)

and, similarly, *H*_B_(*q*, *h*) is given by

*H_B_*(*q*, *h*) = *A*_0_*A*_1_ sinh*q*_1_*h* sinh(*q*_1_(1 − *h*)) sin*q*_2_ − sinh*q*_1_ sin*q*_2_*h* sin(*q*_2_(1 − *h*)) + *A*_0_[*F*(*q*) − *F*(*qh*) − *F*(*q*(1 − *h*))] + *A*_1_[*G*(*q*) − *G*(*qh*) − *G*(*q*(1 − *h*))] + *A*_1_^2^[*F*(*qh*) + *F*(*q*(1 − *h*)) − sinh*q*_1_ cos*q*_2_*h* cos(*q*_2_(1 − *h*))] + *A*_0_/*A*_1_[*G*(*qh*) + *G*(*q*(1 − *h*)) − cosh*q*_1_*h* cosh(*q*_1_(1 − *h*)) sin*q*_2_]
(B2)

where *A*_0_ = (*b*/*q*_1_)^2^ − 1, *A*_1_ = *q*_2_/*q*_1_, *A*_2_ = [(*b*/*q*_1_)^2^ + *A*_1_^2^]/*A*_0_, *F*(*z*) = sinh*z*_1_ cos*z*_2,_ and *G*(*z*) = cosh*z*_1_ sin*z*_2_.

The position function α(*h_j_*^*^, γ_0_), obtained by a perturbation technique and used in Equation (6), reads for the cantilever

α(*h_j_*^*^, γ_0_) = − *H*_C_(*q*_0_, *h_j_*^*^)/[4*A*_1_^4^*H*_DC_(γ_0_)]
(B3)

and for the suspended beam α(*h_j_*^*^, γ_0_) is

α*_i_*(*h_i_*^*^, γ_0_) = − *H*_D*i*_(*q*_0_, *h_i_*^*^)/[4*A*_1_*H*_DT_(γ_0_)]
(B4)

where *H*_DC_(γ_0_) = 2*b*^4^/(*A*_4_*A*_5_) (1 − cosh*q*_1_ cos*q*_2_) − 2(2*q*_1_/*A*_4_ + *A*_4_/*A*_5_) (cosh*q*_1_ cos*q*_2_ + 1) − [1 + *b*^2^/(2*q*_2_^2^) + *b*^4^/(2*q*_1_*A*_5_)] sinh*q*_1_ cos*q*_2_ + [1/*A*_1_ − *b*^2^/(2*q*_1_*q*_2_) + *b*^4^/(2*q*_2_*A*_5_)] cosh*q*_1_ sin*q*_2_ − [*A*_4_/(*q*_1_*q*_2_*A*_5_) + 4/(*q*_2_*A*_4_)] sinh*q*_1_ sin*q*_2_ and *H*_DT_(γ_0_) = [(2*q*_1_^2^ + *b*^2^)/(2*q*_1_*q*_2_)]*G*(*z*_0_) + *b*^2^(3*q*_1_^2^ + *q*_2_^2^)/*A*_3_ sinh*q*_1_ sin*q*_2_ − [(2*q*_2_^2^ − *b*^2^)/(2*q*_2_^2^)]*F*(*z*_0_) − [(8*q*_1_^2^*q*_2_^2^ + *q*_1_^4^ + 3*q*_2_^4^)/*A*_3_] (cosh*q*_1_ cos*q*_2_ − 1)]; *A*_3_ = *q*_1_^2^*q*_2_(*q*_1_^2^ + *q*_2_^2^), *A*_4_ = *q*_1_^2^ + *q*_2_^2^ and *A*_5_(*q*) = *q*_1_*q*_2_^2^.

Similarly, for the suspended beam without tension, the position function α(*h*^*^, γ_0__B_) reads

(B5) $$\alpha (h^{*},\;\gamma_{\text{0B}})=\frac{H(\gamma_{\text{0B}})-H\;[\gamma_{\text{0B}}(1-h^{*})]-H(\gamma_{\text{0B}}h^{*})+\sinh\gamma_{\text{0B}}\sin(\gamma_{\text{0B}}h^{*})\sin[\gamma_{\text{0B}}(1-h^{*})]+\sinh(\gamma_{\text{0B}}h^{*})\sinh[\gamma_{\text{0B}}(1-h^{*})]\sin\gamma_{\text{0B}}}{2H(\gamma_{\text{0B}})}$$

and for the cantilever, α(*h*^*^, γ_0B_) is given by

(B6) $$\alpha (h^{*},\;\gamma_{\text{0B}})=\frac{H(\gamma_{\text{0B}}h^{*})-H\;[\gamma_{\text{0B}}(1-h^{*})]+\sinh\gamma_{\text{0B}}\cos(\gamma_{\text{0B}}h^{*})\cos[\gamma_{\text{0B}}(1-h^{*})]-\cosh(\gamma_{\text{0B}}h^{*})\cosh[\gamma_{\text{0B}}(1-h^{*})]\sin\gamma_{\text{0B}}}{2H(\gamma_{\text{0B}})}$$

where *H*(*z*) = *F*(*z*) − *G*(*z*).
